# mGluR5 as a Potential Orchestrator of Astrocyte Interactions in Neurological Disorders

**DOI:** 10.1155/np/7259018

**Published:** 2025-09-09

**Authors:** Jeongseop Kim, Jiyong Lee, Hyein Song, Ja Wook Koo, Shinwoo Kang

**Affiliations:** ^1^Emotion, Cognition and Behavior Research Group, Korea Brain Research Institute, Daegu, Republic of Korea; ^2^Department of Pharmacology, School of Medicine, Daegu Catholic University, Daegu 42472, Republic of Korea; ^3^Department of Pharmacology, College of Medicine, Soonchunhyang University, Cheonan-si, Republic of Korea; ^4^Department of Brain and Cognitive Sciences, Daegu Gyeongbuk Institute of Science and Technology (DGIST), Daegu 42988, Republic of Korea

**Keywords:** astrocyte, mGluR5, neuroinflammation, synaptic homeostasis, therapeutic potential

## Abstract

Astrocytes and metabotropic glutamate receptor 5 (mGluR5) have emerged as pivotal regulators of synaptic homeostasis and neural communication within the central nervous system (CNS). Although mGluR5 has long been considered neuron-specific, its functional expression in astrocytes is now recognized as essential for calcium (Ca^2+^) signaling, gliotransmission, and the modulation of synaptic plasticity. Dysregulation of astrocytic mGluR5 is increasingly implicated in the pathophysiology of neurodegenerative and psychiatric disorders including Alzheimer's disease (AD), Parkinson's disease (PD), depression, anxiety, and schizophrenia (SCZ) by promoting neuroinflammation, excitotoxicity, and synaptic dysfunction. In this review, we explore the emerging role of astrocytic mGluR5 in mediating astrocyte-neuron communication and its maladaptive regulation in disease contexts. We also assess the therapeutic potential of targeting astrocytic mGluR5, highlighting advances in pharmacological modulators, gene therapy, and RNA-based strategies aimed at restoring homeostatic function. Despite recent progress, critical knowledge gaps remain, particularly regarding the regional specificity of astrocytic mGluR5 effects, its crosstalk with other signaling pathways, and its contribution to chronic neuroinflammation. Addressing these challenges may unlock innovative astrocyte-targeted therapies to restore synaptic integrity and protect against neurodegeneration in CNS disorders.

## 1. Introduction

### 1.1. mGluR5 in Synaptic Modulation and Disease

Metabotropic glutamate receptor 5 (mGluR5), a G-protein-coupled receptor (GPCR), plays a crucial role in synaptic plasticity and maintaining neurological health. As a key modulator of glutamatergic neurotransmission, mGluR5 regulates neuronal excitability and cognitive functions, particularly within the hippocampus (HPC), striatum, and cortical regions essential for emotion, learning, and memory [1]. In contrast to ionotropic receptors that mediate rapid synaptic transmission, mGluR5 activates intracellular signaling cascades that exert long-lasting influences on synaptic strength [2].

**Figure 1:**
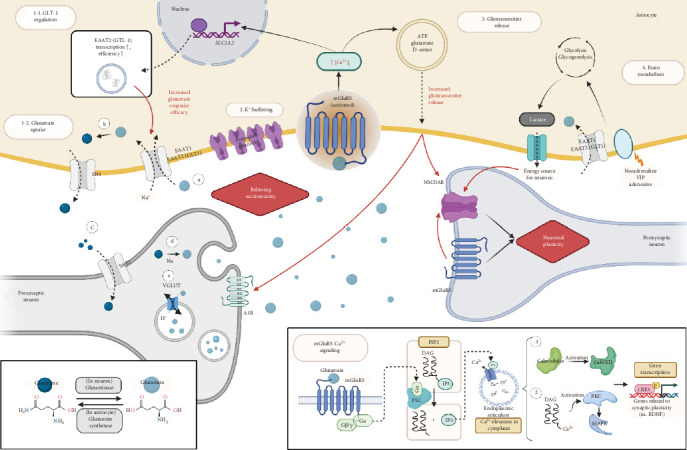
Roles of astrocytic mGluR5 in tripartite synapse astrocytic mGluR5 plays a pivotal role in maintaining synaptic homeostasis primarily by two mechanisms, relieving excitotoxicity and modulating neuronal plasticity. mGluR5 is activated by glutamate, activating PLC enzyme that cleaves PIP2 into DAG and IP3. IP3 increases intracellular Ca^2+^ level, which in turn activates Ca^2+^-dependent enzymes such as CamKII which plays a crucial role in synaptic plasticity, learning, and memory. Ca^2+^ also binds to DAG and activate PKC and MAPK, finally expressing genes like *BDNF*. The process includes NMDAR, another crucial glutamate receptor for neuronal plasticity, LTP, and LTD. Elevated intracellular Ca^2+^ also promotes gliotransmitters (ATP, glutamate, and D-serine) release which interact with presynaptic A1R and NMDAR. In addition, Ca^2+^ enhances GLT-1 transcription and transporter efficiency, thus, regulating glutamate reuptake to relieve excitotoxicity.

mGluR5 belongs to the group I mGluRs, which activate key processes such as long-term potentiation (LTP) and long-term depression (LTD), essential for neurodevelopment, learning, and memory [3, 4]. Upon glutamate binding, group I mGluRs activate phospholipase C (PLC), which in turn produces inositol 1,4,5-trisphosphate (IP3) and prompts the release of intracellular calcium ions (Ca^2+^). Ca^2+^, a crucial second messenger in the central nervous system (CNS), facilitates synaptic plasticity by activating various enzymes such as Ca^2+^/calmodulin-dependent protein kinase II (CaMKII) [2]. Additionally, Ca^2+^ binds to the remaining membrane-bound diacylglycerol (DAG), activating protein kinase C (PKC), which ultimately influences mitogen-activated protein kinase (MAPK) and cyclic AMP response element-binding protein (CREB). This cascade leads to the transcription of various genes involved in synaptic plasticity [5] (Figure 1).

Numerous studies suggest potential interaction between group I mGluRs and ionotropic GluRs, such as N-methyl-D-aspartate receptors (NMDARs), further emphasizing their role in synaptic strengthening and neuronal communication [6–9]. NMDARs may be coactive or not with mGluR5 depending on the context, where the NMDAR subtype and developmental stage play critical roles, thus, enabling intricate synaptic mechanisms that allow both LTP and NMDAR-independent LTD [2].

Dysregulation of mGluR5 signaling has been implicated in several neurological and psychiatric disorders, including Alzheimer's disease (AD), schizophrenia (SCZ), and fragile X syndrome [10, 11]. Overstimulation of mGluR5 activity can lead to excitotoxicity, in which excessive glutamate stimulation causes sustained Ca^2+^ influx and mitochondrial dysfunction, ultimately resulting in neuronal death and disease progression. When Ca^2+^ is excessively released due to mGluR5 overactivation, the intracellular Ca^2+^ regulatory mechanism, mediated by the plasma membrane Ca^2+^ ATPase (PMCA) and the plasma membrane Na^+^/Ca^2+^ exchanger (NCX), becomes overloaded [12]. The excessive Ca^2+^ then induces cellular toxicity by impairing mitochondrial electron transport chain function and generating reactive oxygen species (ROS), leading to neurite degeneration [13]. Consequently, mGluR5 has emerged as a promising therapeutic target, with positive and negative allosteric modulators (PAMs and NAMs) under investigation. For instance, NAMs such as 2-methyl-6-(phenylethynyl)pyridine (MPEP) and basimglurant have shown potential in preclinical studies (Table 1). However, despite these encouraging findings, most mGluR5 modulators have faced limited success in clinical trials, with few progressing beyond phase 1 or 2 (Table 1).

As research continues to elucidate mGluR5's diverse roles across neural networks, its potential as a therapeutic target for various neurodegenerative and psychiatric disorders becomes increasingly apparent.

### 1.2. Astrocytes in Gliotransmission and Neuron Communication: An Overview

Astrocytes, once considered merely support cells, are now recognized as dynamic regulators of brain function [36, 37]. The concept of the tripartite synapse, which includes astrocytes and neuronal pre and postsynaptic elements, highlights their central role in synaptic regulation. These glial cells play a crucial role in maintaining synaptic homeostasis, regulating neurotransmitter levels, and supporting neuronal health. Astrocytes contribute to neurovascular coupling, preserve blood-brain barrier (BBB) integrity, and regulate synaptic activity through potassium buffering and glutamate clearance via EAATs [38–40].

In addition to homeostatic roles, astrocytes contribute to brain metabolism by coupling the neuronal and vascular systems, thereby, distributing nutrients to synaptic components. Both glycolysis, mediated by glutamate uptake, and glycogenolysis, promoted by neuromodulators such as noradrenaline, vasoactive intestinal polypeptide (VIP), and adenosine, result in lactate release by astrocytes. Lactate is then transported to neurons to serve as a key energy source for the brain, while generating reduced nicotinamide adenine dinucleotide (NADH), which modulates NMDAR. This process leads to the expression of genes involved in synaptic plasticity, such as the activity-regulated cytoskeletal (*Arc*) gene and brain-derived neurotrophic factor (*BDNF*) [41, 42] (Figure 1).

Astrocytes, as principal regulators of the BBB, utilize end-foot processes to release signaling molecules such as transforming growth factor-β (TGF-β), which adjusts BBB permeability, particularly during injury or disease [38]. This regulatory function is crucial for maintaining the brain's internal environment, shielding it from toxins while allowing the selective entry of essential nutrients. Astrocytes also regulate extracellular potassium concentrations through Kir4.1 channels, preventing neuronal hyperexcitability, and ensuring stable firing patterns [40].

A key role played by astrocytes is the uptake of excess neurotransmitters such as glutamate from the synaptic cleft. Excitatory amino acid transporter 1 (EAAT1) and EAAT2, also known as glutamate transporter-1 (GLT-1) in astrocytes, act to reabsorb glutamate, thereby, enabling intracellular glutamine synthesis. Glutamine is then transported to neurons via the astrocytic system N transporter 1 (SN1) and the neuronal system A transporter 2 (SAT2), metabolized into glutamate, and captured inside synaptic vesicles (Figure 1).

As astrocytes are increasingly recognized for their role in neuron-glial communication, targeting these cells presents a promising therapeutic avenue for treating conditions such as AD and SCZ. The multifaceted roles of astrocytes, ranging from BBB regulation to neurotransmitter clearance and synaptic modulation, underscore their importance in maintaining brain homeostasis and offer novel targets for treating neurodegenerative and psychiatric disorders [43–45].

### 1.3. Pathophysiological Mechanisms Involving Astrocytic mGluR5 as a Key Trigger

Although the neuronal expression of mGluR5 has been well-documented, with crucial roles in postsynaptic regulation [46, 47], recent findings have highlighted its expression in astrocytes, extending its influence beyond neurons [48] (Figure 1).

Astrocytic mGluR5, located in the perisynaptic space, modulates Ca^2+^ signaling cascades that leads to the propagation of Ca^2+^ waves through gap junctions and the subsequent release of gliotransmitters such as ATP, glutamate, and D-serine agents [49–52]. These gliotransmitters modulate nearby neuronal synapses, influencing synaptic plasticity processes such as LTP and LTD, both of which are essential for cognitive functions like learning and memory [53–55]. Additionally, astrocytic mGluR5 ensures the balance necessary for stable synaptic environments by aiding modulation of other receptors, such as NMDARs and presynaptic adenosine A1 receptors, with the gliotransmitters [55, 56]. Overactivated mGluR5 signaling can lead to dysregulated Ca^2+^ waves and gliotransmitter release, thereby altering astrocyte-mediated glutamate and GABA balance and ultimately causing excitotoxicity. In epilepsy, excessive mGluR5 activity can amplify glutamate spillover and excitotoxicity, while in SCZ, impaired astrocytic mGluR5 function may result in reduced GABAergic tone and hyperactive neuronal networks [11, 57].

mGluR5 and its encoding gene, *GRM5*, are highly expressed in developing immature astrocytes but decline to low levels following astrocytic maturation [58], suggesting that the initial high expression of mGluR5 plays a pivotal role in regulating astrocyte growth and branching [59, 60]. Recent research has expanded the role of mGluR5 by highlighting its reemergence in astrocytes under specific conditions. For example, during neuropathic pain [49] induction or in certain disease states such as AD [61–63] and brain ischemia [61], mGluR5 expression is reactivated in astrocytes, triggering excessive synapse formation and network rewiring. This reemergence can lead to maladaptive synaptic plasticity [49], suggesting a potential role for astrocytic mGluR5 in the development of neurological disorders. Further studies are needed to explore how these findings could translate into therapeutic strategies for neurodegenerative and psychiatric diseases.

The uptake of excess neurotransmitters, particularly glutamate, happens via EAAT1 and EAAT2 (GLT-1), which is tightly regulated by astrocytic mGluR5 [64]. The receptor modulates GLT-1 expression and glutamate uptake to prevent excitotoxicity and maintain excitatory neurotransmission within physiological limits. mGluR5 activation induces Ca^2+^ signaling in astrocytes, driving GLT-1's quantitative and functional upregulation [65, 66] (Figure 1). Notably, fragile X mental retardation protein (FMRP) facilitates this process by binding to mGluR5 mRNA and enhancing its translation. Loss of FMRP disrupts mGluR5 expression and activity, resulting in reduced GLT-1 expression, impaired glutamate uptake, and increased neuronal excitability [67]. Disruption of this system in neurodegenerative diseases can impair glutamate clearance, contributing to excitotoxicity and neuronal damage [68]. In AD, for instance, astrocytic failure to clear synaptic glutamate exacerbates amyloid-beta (Aβ)-induced neurotoxicity [69].

### 1.4. The Objective of the Review

Astrocytic mGluR5 is emerging as a key regulator in neurodegenerative and psychiatric diseases, where its dysregulation contributes to neuroinflammation and synaptic dysfunction. Although the role of neuronal mGluR5 has been extensively studied, the functions of astrocytic mGluR5, which are vital for synaptic homeostasis and the control of neuroinflammatory processes, still need to be explored. Emerging evidence links astrocytic mGluR5 to essential functions, such as Ca^2+^ signaling and gliotransmission, which influence neuron-astrocyte communication and broader CNS regulation [51, 70].

This review explores the distinct roles of astrocytic mGluR5 in disease pathophysiology, and its potential as a therapeutic target. Remarks from studies targeting nonspecific or region-wide mGluR5 are also included in this review if they provide significant insights into astrocyte-neuron interactions or hold value for future research specifically focused on astrocytes. By modulating astrocytic mGluR5 activity, novel strategies may emerge to restore synaptic balance, reduce neuroinflammation, and offer innovative treatments for conditions like AD, depression, and SCZ [11, 71].

## 2. Astrocytic mGluR5 Dysfunction in Neurodegenerative Diseases

Astrocytes, once considered purely supportive, are now recognized for their crucial role in maintaining brain homeostasis and modulating neuronal activity. Under pathological conditions, astrocytes become reactive, adopting neuroinflammatory phenotypes that contribute to the progression of neurodegenerative diseases such as AD and Parkinson's Disease (PD) [36, 72]. Astrocytic mGluR5 regulates these inflammatory responses [73]. While mGluR5 typically supports synaptic homeostasis and neuroprotection under physiological conditions, its dysregulation can trigger inflammatory cascades, exacerbating neuronal damage and disease progression [74].

### 2.1. AD

AD is the most common form of dementia, characterized by progressive memory loss, cognitive decline, and eventual neuronal death. Its pathological hallmarks of AD include the extracellular accumulation of Aβ plaques and the intracellular formation of neurofibrillary tangles composed of hyperphosphorylated tau protein. These abnormalities disrupt synaptic integrity, impair neuronal communication, and drive neurodegeneration [75].

**Figure 2:**
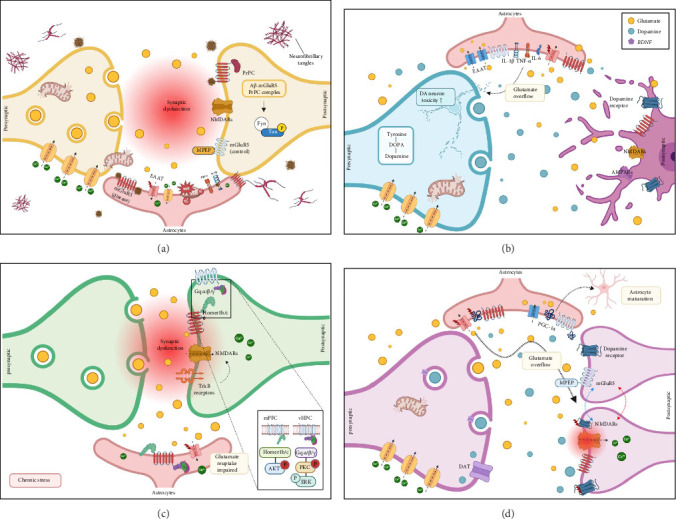
The Role of mGluR5 in neurodegenerative and psychiatric disorders. (A) Alzheimer's disease (AD): in disease states, astrocytic mGluR5 interacts with Aβ oligomers, triggering neuroinflammation via production of IL-1β, TNF-α, and oxidative stress, and impairing EAAT function, which contributes to glutamate overflow and synaptic dysfunction. Postsynaptic neuronal mGluR5 forms a complex with Aβ-PrPC to activate Fyn kinase, leading to tau hyperphosphorylation and NMDA receptor dysfunction. (B) Parkinson's disease (PD): dysregulated astrocytic mGluR5 elevates IL-6, IL-1β, and TNF-α production, reduces EAAT expression, thereby, exacerbating dopaminergic neuronal toxicity and impairing glutamate balance. (C) Depression: chronic stress suppresses astrocytic mGluR5-EAAT2 signaling, disrupting glutamate clearance. Postsynaptically, altered interactions between mGluR5 and Homer1b/c impair NMDA receptors and TrkB signaling, amplifying excitotoxicity, and reducing synaptic plasticity. (D) Schizophrenia: aberrant astrocytic mGluR5 signaling reduces PGC-1α mediated mitochondrial biogenesis, disturbing astrocyte maturation and glutamate regulation. Neuronal mGluR5 downregulates NMDA receptor activity, while mGluR5 antagonists such as MPEP may restore cognitive function and normalize glutamatergic signaling.

A growing body of evidence implicates mGluR5 in AD progression, mainly through its interactions with Aβ oligomers (Figure 2). Astrocytes, key glial cells involved in maintaining neuronal homeostasis, modulate mGluR5 activity. Under physiological conditions, astrocytic mGluR5 regulates neurotransmitter levels and protects against excitotoxicity. However, in AD, this regulation becomes disrupted, particularly in response to Aβ accumulation. Aβ, especially at a concentration of 100 nM Aβ42, has been shown to increase astrocytic Ca^2+^ levels, potentially through a mechanism in which Aβ forms channel-like structures that facilitate Ca^2+^ entry. This Ca^2+^ increase was observed several minutes after Aβ treatment in isolated astrocytes in vitro, suggesting that activation of the intracellular astrocytic signaling mechanism requires sufficient delay [76]. Consequently, calcineurin (CaN) becomes activated, which in turn promotes mGluR5 overexpression. This may further elevate intracellular Ca^2+^ levels, establishing a vicious cycle of sustained signaling [10, 76, 77].

This overexpressed mGluR5, along with elevated intracellular Ca^2+^ levels, impairs Aβ clearance and promotes chronic neuroinflammation. In this state, astrocytes release pro-inflammatory cytokines such as IL-1β, TNF-α, and ROS, further contributing to neuronal damage [78]. Excessive mGluR5 signaling in astrocytes also enhances nitric oxide (NO) production and oxidative stress, aggravating synaptic dysfunction and neurotoxicity [79, 80].

In neurons, mGluR5 interacts with Aβ oligomers through a prion protein (PrPC)-mediated mechanism, forming a complex that activates nonreceptor tyrosine kinase Fyn, a key enzyme involved in tau phosphorylation and NMDAR internalization. This signaling cascade contributes to synaptic deterioration, impaired plasticity, and cognitive deficits associated with AD [81]. Moreover, mGluR5 overactivation amplifies glutamatergic transmission, promoting excitotoxicity [82]. Collectively, this pathological feedback loop-comprising Aβ accumulation, excitotoxicity, and inflammation, accelerates neurodegeneration in AD.

Targeting mGluR5 has emerged as a promising therapeutic strategy for AD. Pharmacological inhibition of mGluR5 disrupts the Aβ-mGluR5-PrPC complex, thereby, attenuating downstream signaling cascades involving Fyn activation, tau hyperphosphorylation, and synaptic degeneration. However, it is important to note that commonly used pharmacological agents such as MPEP and fenobam, are not selective for astrocytic mGluR5 and also target neuronal mGluR5. This lack of cell-type specificity may contribute to variable therapeutic outcomes and highlights the need for more targeted approaches that distinguish between astrocytic and neuronal mGluR5 signaling. Despite these limitations, mGluR5 antagonists have shown efficacy in preclinical AD models by preventing cognitive decline, restoring synaptic function, and reducing neuroinflammation [10, 81]. In addition, these compounds alleviate oxidative stress and suppress the production of proinflammatory cytokines, offering the dual benefit of mitigating both excitotoxicity and inflammation [11, 83].

Given the role of mGluR5 in neurons and astrocytes, modulating its activity represents a multifaceted therapeutic approach for AD. By disrupting toxic Aβ interactions, reducing excitotoxicity, and alleviating neuroinflammation, mGluR5 inhibition may confer neuroprotection and potentially slow the progression of AD [84]. However, as previous PET imaging studies have reported reduced mGluR5 levels in the HPC of AD patients (Table 2), further clinical investigations are warranted to determine the long-term efficacy and optimal timing of mGluR5-targeted therapies in humans.

### 2.2. PD

PD is primarily characterized by motor symptoms such as bradykinesia, tremors, muscular rigidity, and postural instability, which result from the progressive loss of dopaminergic neurons in the substantia nigra pars compacta. These neurons play a critical role in regulating movement by modulating basal ganglia circuits [99]. However, the clinical manifestations of PD extend beyond motor dysfunction and include a spectrum of nonmotor symptoms such as cognitive decline, depression, and sleep disturbances that significantly impact patients' quality of life [100]. In recent years, a growing body of evidence suggests that PD pathogenesis involves not only dopaminergic degeneration but also significant alterations in glutamate transmission and neuroinflammatory processes (Figure 2).

Although few studies have investigated astrocytic mGluR5 in PD, overactivated astrocytic mGluR5 signaling plays a crucial role in PD by promoting the production of neuroinflammatory mediators, which impair the clearance of excess glutamate. This failure to regulate extracellular glutamate levels leads to its accumulation, thereby, driving excitotoxicity [101]. Such dysregulation is particularly detrimental to dopaminergic neurons, which are highly vulnerable to glutamate-induced excitotoxic stress [45]. As a result, dysfunctional astrocytic mGluR5 signaling not only perpetuates neuroinflammation but also fails to safeguard neurons from excitotoxic damage, contributing to the self-reinforcing cycle of neurodegeneration characteristic of PD.

Group I mGluRs, including mGluR1 and mGluR5, is critical for the basal ganglia by modulating excitatory neurotransmission and synaptic plasticity. Activation of these receptors at multiple sites within the basal ganglia circuitry can contribute to the synaptic overactivity observed in PD [102]. In PD, the degeneration of dopaminergic neurons disrupts the balance between excitatory and inhibitory signaling in the striatum, leading to increased glutamate release and subsequent overactivation of mGluR5 [103]. This aberrant activation promotes excitotoxicity, oxidative stress, and mitochondrial dysfunction, thereby, exacerbating neuronal loss and worsening motor symptoms [100]. In parallel, reactive astrocytes in both the substantia nigra and striatum secrete proinflammatory cytokines including TNF-α, IL-1β, and IL-6, which amplify neuroinflammation and contribute to the progressive degeneration of dopaminergic neurons [104].

Beyond motor dysfunction, aberrant mGluR5 activity has also been implicated in nonmotor symptoms of PD, including cognitive decline and mood disorders. Overactivation of mGluR5 and subsequent excitotoxicity in brain regions such as the prefrontal cortex (PFC) and HPC, key centers for cognition and emotion, impair synaptic plasticity and neurotransmission, thereby, worsening cognitive decline and mood disorders in PD patients [103].

Therapeutic strategies targeting mGluR5 have shown promise in alleviating both motor and nonmotor PD symptoms. mGluR5 antagonists, such as MPEP and AFQ056, have been shown to reduce neuroinflammation, protect dopaminergic neurons, and restore glutamate homeostasis [103, 105]. However, these compounds, do not selectively target astrocytic mGluR5 and are also known to modulate neuronal signaling. This pharmacological limitation should be carefully considered when interpreting both preclinical and clinical outcomes. Notably, mGluR5 NAMs including AFQ056, have demonstrated beneficial effects in clinical trials by improving motor symptoms in PD patients experiencing levodopa-induced dyskinesias (Table 1). Beyond motor improvements, these agents may also ameliorate cognitive and affective disturbances by restoring glutamatergic balance in the PFC and HPC [94].

Given the broad expression of mGluR5 in both neurons and astrocytes, targeting this receptor represents a comprehensive therapeutic approach for PD. Inhibiting mGluR5 may simultaneously mitigate excitotoxicity, preserve dopaminergic neurons, suppress neuroinflammation, and improve cognitive as well as affective symptoms.

### 2.3. Pain

Neuropathic pain is a chronic condition caused by injury or dysfunction of the somatosensory nervous system, characterized by heightened sensitivity to stimuli (allodynia) and spontaneous pain. Although traditionally associated with neuronal hyperexcitability and spinal sensitization, recent evidence suggests a pivotal role for astrocytes in the development and maintenance of neuropathic pain [49, 106].

Astrocytic mGluR5 has emerged as a key modulator in this context. Although, typically downregulated in mature astrocytes, mGluR5 is transiently re-expressed in astrocytes of the somatosensory cortex following peripheral nerve injury. This reactivation induces robust Ca^2+^ signaling and upregulation of multiple synaptogenic factors, including thrombospondin-1, glypican-4, and hevin, which together promote excessive excitatory synaptogenesis and network hyperexcitability. A previous study demonstrated that selective deletion of astrocytic mGluR5 in the somatosensory cortex abolished the development of mechanical allodynia in mice, indicating a direct causal role in chronic pain [49, 106].

Importantly, the upregulation of mGluR5 in astrocytes preceded the inset of pain hypersensitivity, suggesting a causative rather than compensatory role. Astrocytic mGluR5 activation led to structural synaptic remodeling that shifted the excitation/inhibition balance toward hyperexcitability, thereby, sustaining persistent pain states. Complementary evidence from the anterior cingulate cortex also highlights the contribution of astrocytic mGluR5 to chronic pain. In this region, injury-induced astrocyte hyperactivity has been shown to elevate mGluR5-dependent glutamate release, leading to heightened excitatory transmission and persistent pain signaling. Suppressing astrocytic mGluR5 activity in the ACC effectively restored synaptic balance and reduced pain behaviors [106]. Together, these findings underscore the therapeutic potential of targeting astrocytic mGluR5 in neuropathic pain, expanding its relevance beyond neurodegenerative and psychiatric diseases.

## 3. Astrocytic mGluR5 Dysfunction in Psychiatric Disorders

Targeting astrocytic mGluR5 is emerging as a promising therapeutic approach for addressing psychiatric disorders. Dysregulation of astrocytic mGluR5 has been associated with mood, cognitive, and behavioral dysfunctions in conditions like depression, anxiety, SCZ, and autism [107]. Modulating its activity may help restore synaptic balance, reduce neuroinflammation, and enhance neuroplasticity. Various pharmacological agents, including mGluR5 antagonists or modulators, are currently under investigation for their ability to counteract maladaptive astrocytes-neurons interactions implicated in these disorders [108–110].

### 3.1. Depression

Major depressive disorder (MDD) is marked by persistent mood disturbances and cognitive impairments, often associated with disruptions in balance between excitatory and inhibitory neurotransmission [111].

Dysregulation of astrocytic mGluR5 contributes to this imbalance by affecting extracellular glutamate levels, synaptic plasticity, and neuroinflammatory responses. Chronic stress, a major risk factor for MDD, has been shown to downregulates astrocytic mGluR5 in brain regions essential for emotional regulation, such as the PFC, HPC, and amygdala [112]. Conversely, other studies using human and rodent MDD models have reported increased astrocyte reactivity and upregulation of astrocytic markers in some brain regions [113]. This upregulation may result in excessive glutamate release, synaptic hyperexcitability, and neurotoxicity, hallmarks of MDD pathology (Figure 2).

Astrocytes play a central role in glutamate clearance via EAATs, and chronic stress impairs this function, further intensifying excitotoxicity and neuroinflammation [39]. Astrocytic mGluR5 also regulates astrocytic Ca^2+^ signaling, which is critical for synaptic signaling and gliotransmission [114]. Under chronic stress, mGluR5 dysfunction disrupts Ca^2+^ homeostasis, diminishing neurotrophic support, and worsening synaptic deficits associated with depression [115].

Chronic stress disrupts the cellular mechanisms essential for maintaining protein homeostasis in response to misfolded proteins. The accumulation of misfolded proteins in the endoplasmic reticulum (ER) activates signaling pathways such as PERK/eIF2, which are involved in mGluR5-dependent LTD and memory decline [116]. Additionally, chronic stress inhibits CREB phosphorylation and selectively downregulates *BDNF* expression in the HPC, leading to the internalization of AMPARs and resulting in depression-like behaviors [116, 117]. This impaired astrocyte-neuron communication may also lower *BDNF* levels, increasing susceptibility to stress-induced deficits in neuroplasticity [118].

The dual role of astrocytic mGluR5, shifting between neuroprotection and neurotoxicity, complicates its functional interpretation [119, 120]. Under normal conditions, mGluR5 supports synaptic plasticity through Ca^2+^ signaling; however, in chronic stress-induced overactivation, it leads to neuroinflammation and synaptic dysfunction. Pharmacological studies targeting mGluR5 have shown therapeutic potential, with antagonists like 3- ([2-Methyl-1,3-thiazol-4-yl]ethynyl)pyridine (MTEP) and basimglurant reducing depressive-like behaviors by restoring synaptic homeostasis and mitigating glutamatergic hyperactivity [121]. It should be emphasized that these drugs are not selective for astrocytic mGluR5 and concurrently affect neuronal receptors, which may confound the interpretation of behavioral and molecular outcomes in experimental models. Furthermore, chronic stress downregulates astrocytic mGluR5 in the HPC, mirroring the synaptic deficits observed in depression, while overexpression of mGluR5 reverses these deficits [112]. An interesting aspect of previous clinical PET studies is the complex results observed in mGluR5 levels (Table 2). This complexity likely reflects the heterogeneous nature of depression. For example, mGluR5 levels were found to be notably decreased in the PFC of nonsmoking persons or drug-naïve young adults (Table 2). Additionally, clinical trials using mGluR5 antagonists and NAMs have not shown significant differences, but some evidence suggests that these agents, when combined with conventional antidepressants, may offer additional benefits (Table 1). Consequently, the role of mGluR5 in depression is highly complex and warrants further investigation.

The interaction between mGluR5 and Homer1 proteins further underscores the receptor's role in depression. Homer1b/c, downregulated in the PFC during MDD, especially in projections from the basolateral amygdala, modulates mGluR5 signaling, impacting early neurodevelopment and synaptic organization [122]. Homer1a, an activity-dependent splice variant, disrupts Homer1b/c-mGluR5 interactions, enabling signaling plasticity but potentially destabilizing ER-plasma membrane microdomains critical for Ca^2+^ signaling and glutamate regulation [123]. Excessive Homer1a expression in reactive astrocytes disrupts these microdomains, potentially contributing to the synaptic dysfunction observed in depression [123].

Recent RNA sequencing studies confirm that Homer1 is expressed in cortical astrocytes, with Homer1b/c and mGluR5 forming clusters within astrocytic processes, similar to their neuronal distribution [124, 125]. These microdomains function as astrocytic signaling hubs, regulating synaptic plasticity and glutamate homeostasis. In pathological conditions such as depression, the altered Homer1a expression has been implicated in synaptic dysfunction. Increased Homer1a levels may disrupt synaptic organization, potentially impairing mGluR5 function and glutamate clearance, which could contribute to MDD-related synaptic deficits [126].

In summary, Homer1 proteins are critical regulators of mGluR5 signaling in neurons and astrocytes, balancing structural stability and signaling adaptability. Dysregulation of the mGluR5-Homer1 interaction contributes to the synaptic and neuroinflammatory pathology of MDD. Targeting this interaction offers a promising strategy for restoring proper glutamate signaling and addressing treatment-resistant depression.

### 3.2. Anxiety

Anxiety disorders, including posttraumatic stress disorder (PTSD), are characterized by heightened fear responses and persistent worry, often attributed to dysregulation in glutamatergic transmission and synaptic plasticity [127, 128].

mGluR5 plays a pivotal role in the regulation of anxiety, particularly within the amygdala, a central hub for fear conditioning and emotional learning [129]. Hyperactivation of mGluR5 in the amygdala has been closely associated with exaggerated fear responses and anxiety-like behaviors in preclinical models [128, 130]. Additionally, mGluR5 signaling in the PFC and HPC plays a significant role in regulating stress responses, with excessive receptor activation promoting neuronal hyperexcitability and dysregulated fear memory processing [131]. Notably, previous studies using mGluR5 radioligand-based PET imaging have shown increased mGluR5 levels in the PFC and HPC of PTSD patients (Table 2), further supporting the involvement of mGluR5 in the pathophysiology of anxiety disorders.

Pharmacological inhibition of mGluR5 has demonstrated substantial efficacy in preclinical models of anxiety. Antagonists like MPEP and fenobam reduce anxiety-like behaviors by mitigating glutamatergic hyperactivity in the amygdala and prefrontal circuits [128, 132]. Interestingly, astrocytes also exhibit abnormal mGluR5 activity in anxiety disorders, which contributes to disrupted glutamate homeostasis and exacerbated neuroinflammatory processes [133]. Therapeutic strategies targeting both neuronal and astrocytic mGluR5 may provide a comprehensive approach to restoring glutamatergic balance and alleviating anxiety symptoms (Table 1).

mGluR5 dynamically regulates glutamate homeostasis through its complex interaction with GLT-1. Acute activation enhances glutamate clearance, whereas chronic overactivation has been associated with downregulation of GLT-1 and GLAST, potentially leading to glutamate accumulation and excitotoxicity in stress-sensitive regions, such as the PFC and HPC [66, 134]. Targeting the mGluR5-GLT-1 axis may, therefore, provide a promising therapeutic strategy to restore glutamatergic homeostasis and mitigate PTSD-related neurobiological dysfunction, as this imbalance is thought to underline key features of PTSD, including cognitive deficits and impaired fear extinction.

Recent animal studies using the inescapable foot shock (IFS) model of PTSD have demonstrated increased mGluR5 expression, alongside reduced levels of *BDNF*, its receptor TrkB, and GLT-1 in both the PFC and HPC [135]. Remarkably, these molecular and behavioral abnormalities were reversed by MPEP administration, underscoring the central role of mGluR5 in mediating glutamate dysregulation under stress conditions.

### 3.3. SCZ

SCZ is marked by positive (hallucinations and delusions), negative (apathy and social withdrawal), and cognitive symptoms [136], with glutamatergic dysfunction, particularly NMDAR hypofunction, strongly implicated in its pathophysiology [137]. mGluR5, a key modulator of NMDAR, plays an essential role in synaptic plasticity and cognitive function, both of which are impaired in SCZ [11, 138]. Reduced mGluR5 activity in the PFC and HPC may contribute to cognitive and negative symptoms by impairing synaptic plasticity and NMDAR function [131] (Figure 2).

In the context of 22q11 deletion syndrome (22q11DS), a major genetic risk factor for SCZ, mitochondrial dysfunction emerges as a critical component. Several genes deleted in 22q11DS encode mitochondrial proteins, linking mitochondrial disturbances to the onset of SCZ [139, 140]. During early development, astrocytic mGluR5 drives mitochondrial biogenesis through PPARγ co-activator-1α (PGC-1α), which is essential for astrocyte maturation and synaptic support [60]. This astrocyte-neuron interplay is vital for functional circuit formation, and disruptions in astrocytic maturation, especially in the late-developing PFC, may underlie cognitive deficits in SCZ [141, 142].

In both SCZ and 22q11DS, astrocyte mediated mitochondrial dysfunction may disrupt synaptogenesis and neural circuit development [143]. Notably, induced pluripotent stem cell (iPSC)-derived neurons from 22q11DS patients without SCZ exhibit compensatory mitochondrial upregulation, while those with SCZ show reduced ATP production [144]. Activating the PGC-1α pathway restores mitochondrial function, emphasizing its protective role against SCZ development in 22q11DS [144]. Thus, astrocytic mGluR5 coordinates synaptic activity with mitochondrial energy production during brain development. Its regulation of PGC-1α-driven mitochondrial biogenesis is vital for maintaining astrocyte activity and synaptic integrity [59, 60]. In SCZ and 22q11DS, dysregulation of this pathway may underlie cognitive and neurodevelopmental impairments. Targeting astrocytic mGluR5 and boosting mitochondrial function could be a promising strategy for reducing SCZ risk in 22q11DS patients. Beyond cognitive functions, mGluR5 also influences the dopaminergic system, which is implicated in the positive symptoms of SCZ. By regulating dopamine in the striatum and PFC, mGluR5 dysregulation contributes to hyperdopaminergic states associated with hallucinations and delusions [145, 146]. Preclinical studies suggest that mGluR5 agonists or PAMs enhance NMDAR function and mitigate cognitive deficits in SCZ models [146, 147] (Table 1), highlighting mGluR5 as a promising therapeutic target. However, human PET studies have not identified significant differences in mGluR5 levels between SCZ patients and controls. Given the limited number of studies in this area (Table 2), further investigation is needed.

## 4. Conclusion and Future Directions

### 4.1. Integration of mGluR5 and Astrocyte Research

Recent breakthroughs have transformed our understanding of astrocytes, highlighting their active involvement in synaptic transmission, neuroinflammation, and plasticity. No longer viewed as passive support cells, astrocytes are now recognized as critical contributors to brain physiology. The discovery that mGluR5, a critical modulator of glutamatergic signaling, is expressed in both neurons and astrocytes has introduced new dimensions to the study of neuron-glia interactions in health and disease [37, 148]. Astrocytic mGluR5 regulates Ca^2+^ signaling, gliotransmission, and neuroinflammatory responses, underscoring its role in synaptic homeostasis and brain plasticity [149].

In pathological states, such as neurodegenerative and psychiatric disorders, dysregulated astrocytic mGluR5 contributes to disease progression through mechanisms like excitotoxicity, neuroinflammation, and synaptic imbalance [150]. Targeting this glial-neuronal crosstalk presents a novel therapeutic strategy, shifting focus beyond traditional neuron-centric approaches to treating brain disorders.

### 4.2. Open Questions

Despite notable advances in the study of astrocytic mGluR5, several critical questions remain unresolved. While its role in mediating Ca^2+^ signaling within astrocytes is well established, the downstream consequences for neuronal circuits, particularly under pathological conditions, are still poorly understood. In disorders such as SCZ and depression, the precise mechanisms through which astrocytic mGluR5 modulates synaptic plasticity and cognitive function remain to be elucidated [115]. This highlights the need for refined studies using astrocyte-specific tools to dissect its contributions across different brain regions and cell populations.

Another central uncertainty concerns the region-specific roles of astrocytic mGluR5. Given its expression in diverse brain regions, each with distinct roles in cognition, motor control, and emotional regulation, its region-dependent contributions to disease pathologies remain poorly characterized [102]. Equally important is the question of disease-stage specificity. Emerging evidence suggests that astrocytic mGluR5 may exert protective effects during early phases of injury or stress, but may become maladaptive when chronically activated. Understanding how its function evolves across the course of disease whether neurodegenerative or psychiatric could help define time-sensitive therapeutic windows and reduce the risk of therapeutic failure due to misaligned treatment timing.

Additionally, the dual role of mGluR5 in neuroinflammation requires deeper investigation. Some studies suggest that acute activation may help contain inflammatory responses, whereas chronic overactivation may exacerbate proinflammatory signaling, glutamate dysregulation, and excitotoxicity. Understanding the molecular switch between these two modes of action could have substantial therapeutic implications, particularly in diseases such as AD or PD, where chronic inflammation is a hallmark.

Finally, astrocytic mGluR5 does not act in isolation. Astrocytes express a wide array of receptors including purinergic, adrenergic, and GABAergic types that may interact with or modulate mGluR5 signaling. The nature of this receptor crosstalk remains largely unexplored. Investigating how these interactions may open new avenues for combination therapies that target multiple glial pathways, thereby, more effectively restoring synaptic and metabolic homeostasis.

Collectively, addressing these open questions will not only refine our understanding of astrocytic mGluR5 function but also accelerate the development of precision therapies tailored to disease context, stage, and regional pathology.

### 4.3. Prospective Therapies

Astrocytic mGluR5 represents a promising therapeutic target across a range of neurological and psychiatric disorders. In neurodegenerative diseases such as AD and PD, mGluR5 antagonists, including MPEP and CTEP, have shown potential to reduce synaptic dysfunction, excitotoxicity, and neuroinflammation [10]. By preventing the overactivation of mGluR5 in reactive astrocytes, these compounds may help preserve synaptic homeostasis and promote neural health. While preclinical studies have yielded encouraging results, further clinical trials are necessary to evaluate their long-term efficacy and safety in human populations.

In psychiatric disorders such as SCZ and depression, modulating astrocytic mGluR5 offers a novel approach to addressing cognitive and emotional impairments. PAMs of mGluR5 agents that enhance receptor activity without causing overexcitation are being investigated for their ability to improve cognition and synaptic plasticity [146]. Compared to direct agonists, PAMs provide more refined modulation, potentially minimizing excitotoxicity risk while enhancing synaptic resilience. Combining mGluR5 targeting agents with conventional antidepressants or antipsychotics could provide a synergistic approach that engages both astrocytic and neuronal mechanisms, thereby, enhancing therapeutic efficacy.

Despite recent progress, a major limitation of current mGluR5 modulators lies in their lack of cell-type specificity. Most pharmacological agents indiscriminately target both astrocytic and neuronal mGluR5, complicating the attribution of observed effects to glial mechanisms and potentially inducing unintended neuronal consequences. To address this, future research should prioritize the development of astrocyte-selective targeting strategies such as viral vectors, nanoparticle-based systems, or astrocyte-specific promoters. These approaches will be essential for precisely targeting astrocytic mGluR5 and translating glia-centered findings into clinical practice.

Emerging genetic techniques, such as gene therapies and RNA-based interventions, offer promising approaches for the selective modulation of astrocytic mGluR5. By employing viral vectors or RNA interference, researchers could precisely regulate mGluR5 expression in specific brain regions, providing a precision medicine approach to disorders like autism spectrum disorder and fragile X syndrome, where mGluR5-mediated plasticity is disrupted [43, 44]. These genetic interventions hold the potential for personalized treatments finely tuned to individual disease profiles.

Importantly, while mGluR5 inhibition is beneficial in certain contexts, activation of this receptor could be advantageous in disorders characterized by synaptic failure and cognitive decline. Therefore, future therapies must be adaptable and capable of either enhancing or inhibiting mGluR5 signaling depending on the disease stage, context, and underlying pathology. Achieving optimal balance between neuroprotection and maintaining physiological astrocytic function will be essential for the successful development of mGluR5-targeted treatments.

## Figures and Tables

**Table 1:** Preclinical and clinical trials of mGluR5 drugs in neurological and psychiatric disorders.

Disorder	Study type	Sample size	Ligand	Main findings	Reference
Major depressive disorder (MDD)	Preclinical study	GRN-529 (Wyeth Pharmaceuticals)	Negative allosteric modulator (NAM)	Dose-dependent efficacy shown by reduced immobility in tail suspension and FST models	Hughes et al. [14]
	Preclinical study	2-Methyl-6-(phenylethynyl)pyridine (MPEP)	Antagonist	Reduced immobility in wild-type mice during FST	Li et al. [15]
	Preclinical study	3-([2-Methyl-4-thiazolyl] ethynyl)pyridine (MTEP)	Antagonist	Reduced immobility during FST	[16]
	Preclinical study	MK-801	Positive allosteric modulator (PAM)	Restores cognitive function by modulating mPFC glutamate levels	LaCrosse et al. [17]
	Preclinical study	DSR-98776	Negative allosteric modulator (NAM)	Produces antidepressant-like actions in rats	Kato et al. [18]
	Clinical study	Basimgluran	Negative allosteric modulator (NAM)	No significant difference in MADRS scores between adjunctive Basimglurant MR and placebo; patient-rated improvements observed at a 1.5 mg dose	Quiroz et al. [19]
	Clinical study	Basimglurant	Negative allosteric modulator (NAM)	Basimglurant (1.5 mg q.d.) demonstrated consistent antidepressant effects when added to SSRI or SNRI therapy, supporting further investigation for depressive disorders	Quiroz et al. [19]
	Clinical study	Basimglurant	Negative allosteric modulator (NAM)	Adjunctive 1.5 mg basimglurant modified-release showed no difference from placebo on the primary MADRS outcome but demonstrated antidepressant effects on secondary patient-rated measures, with good tolerability, warranting further investigation in depressive disorders	Quiroz et al. [19]
	Clinical study	Basimglurant	Negative allosteric modulator (NAM)	Completed phase 1 trials	ClinicalTrials.gov ID NCT00809562
	Clinical study	Basimglurant	Negative allosteric modulator (NAM)	Completed phase 1 trials	ClinicalTrials.gov ID NCT02433093
	Clinical study	Basimglurant	Negative allosteric modulator (NAM)	Completed phase 2 trials	ClinicalTrials.gov ID NCT01437657
	Clinical study	AZD2066	Antagonist	Terminated phase 2 trials	ClinicalTrials.gov ID NCT01145755

PTSD, anxiety-related disorder	Preclinical study	ADX47273	Positive allosteric modulator (PAM)	Enhanced extinction learning during the retrieval-reconsolidation phase and improved reversal learning in the MWM	Xu et al. [20]
	Preclinical study	2-Methyl-6- (phenyl ethynyl)pyridine (MPEP)	Antagonist	Blocks the autonomic response to stress-induced hyperthermia	Spooren et al. [21]
	Preclinical study	CDPPB	Positive allosteric modulator (PAM)	Facilitated extinction of contextual fear memory, enhanced initial fear memory formation, and had no effect on memory retrieval	Sethna and Wang [22]

Schizophrenia-related deficits	Preclinical study	CDPPB	Positive allosteric modulator (PAM)	mGluR5 PAMs enhance hippocampus-dependent spatial learning by modulating LTP and LTD in an activity-specific manner, providing a potential approach for treating cognitive impairments	Ayala et al. [4]
	Preclinical study	CDPPB	Positive allosteric modulator (PAM)	CDPPB attenuated cognitive deficits caused by NMDA receptor hypofunction	Stefani and Moghaddam [23]
	Preclinical study	CDPPB	Positive allosteric modulator (PAM)	Sub-chronic administration of CDPPB improves phencyclidine-induced cognitive deficits in mice	Horio et al. [24]
	Preclinical study	VU0409551, 5PAM523	Positive allosteric modulator (PAM)	VU0409551 demonstrated strong antipsychotic-like and cognition-enhancing effects in animal models	Rook et al. [9]

Parkinson's Disease	Clinical study	Mavoglurant	Negative allosteric modulator (NAM)	Improvement of dyskinesia symptoms and reduction in L-dopa dosage frequency	Petrov et al. [25]
	Clinical study	Dipraglurant	Negative allosteric modulator (NAM)	In a phase 2A double-blind, placebo-controlled trial, dipraglurant significantly reduced peak-dose dyskinesia in Parkinson's disease without worsening parkinsonism, demonstrating safety, tolerability, and efficacy	Tison et al. [26]
	Clinical study	Mavoglurant	Negative allosteric modulator (NAM)	Safe, limited improvement in PD-LID symptoms	Stocchi et al. [27]
	Clinical study	Mavoglurant	Negative allosteric modulator (NAM)	Mavoglurant, combined with higher doses of L-dopa, may effectively treat Parkinson's disease patients with L-dopa-related motor fluctuations and dyskinesias	Kumar et al. [28]
	Clinical study	BMS-984923 (ALX-001)	Silent allosteric modulator (SAM)	Phase 2 clinical development	ClinicalTrials.gov ID NCT05804383
	Preclinical study	MPEP	Antagonist	Full reversal of akinesia at all doses tested	Breysse et al. [29]
	Preclinical study	MPEP	Antagonist	Reversal of akinesia occurs with coadministration of a lower MPEP dose, while individual MPEP administration is only effective at higher doses	Turle-Lorenzo et al. [30]
	Preclinical study	MPEP	Antagonist	Improvement in PD-LID symptoms with coadministration	Morin et al. [31]
	Preclinical study	MTEP	Antagonist	Catalepsy inhibition observed only with 3 and 5 mg/kg MTEP doses at 30- and 60-min postinjection	Ossowska et al. [32]

Alzheimer's disease	Preclinical study	BMS-984923	Silent allosteric modulator (SAM)	SAM binding to mGluR5 does not alter glutamate signaling but significantly reduces its interaction with PrPC bound to Aβo	Haas et al. [33]
	Preclinical study	CDPPB	Positive allosteric modulator (PAM)	CDPPB treatment mitigates Aβ-induced pathological alterations in vitro, in vivo, and in a transgenic mouse model of AD	Bellozi et al. [34]
	Clinical study	BMS-984923 (ALX-001)	Silent allosteric modulator (SAM)	Phase 2 clinical development	ClinicalTrials.gov ID NCT05804383
	Clinical study	11C-ABP688	Antagonist	A substantial 43% decrease in mGluR5 binding in the hippocampus of AD patients, while statistically nonsignificant reduction in the cortical region	Ametamey et al. [35]

**Table 2:** Summary of postmortem and PET studies on mGluR5 in neurological and psychiatric disorders.

Disorder	Study type	Sample size	Ligand	Main findings	Reference
Major depressive disorder (MDD)	PET imaging	11 MDD and 11 HC	[11C] ABP688	Lower mGluR5 binding in multiple areas, including frontal, temporal, and parietal cortices, right thalamus, bilateral insula, left hippocampus, left posterior cingulate cortex, and precentral gyrus	Deschwanden et al. [85]
	PET imaging	20 MDD and 22 HC	[11C] ABP688	No deficit in mGluR5 availability in late-life MDD patients	DeLorenzo et al. [86]
	PET imaging	30 MDD and 35 HC	[18F] FPEB	No significant alterations in mGluR5 volume of distribution in medication-free patients	Abdallah et al. [87]
	PET imaging, rs-fMRI	16 MDD and 15 HC (drug-naïve young adults)	[11C] ABP688	Significantly lower mGluR5 availability in anterior and ventrolateral prefrontal cortex in MDD patients	Kim et al. [88]
	PET imaging	20 MDD and 18 HC (nonsmoking)	[11C] ABP688	Lower mGluR5 availability in bilateral superior, left middle, and inferior frontal cortex in MDD patients with low social avoidance compared to high social avoidance and healthy controls	Kim et al. [89]

Posttraumatic stress disorder (PTSD)	PET imaging	29 PTSD and 29 HC	[18F] FPEB	Increased mGluR5 expression in vmPFC, dlPFC, amygdala, and hippocampus	Davis et al. [90]
	PET imaging	10 PTSD and 10 HC	[18F] FPEB	Increased mGluR5 expression in dlPFC, OFC, and ventral striatum	Holmes et al. [91]
	PET imaging	28 PTSD, 21 MDD, and 28 HC	[18F] FPEB	Study conducted but findings not provided	Esterlis et al. [92]

Schizophrenia-related deficits	PET imaging	15 schizophrenia and 15 HC	[11C] ABP688	The distribution volume ratio showed no difference between individuals with schizophrenia and controls	Akkus et al. [93]

Parkinson's disease	PET imaging	9 PD and 8 HC	[18F] FPEB	Significantly upregulated mGluR5 in striatal and mesotemporal gray matter regions affected by PD	Kang et al. [94]

Alzheimer's disease	PET imaging (in vitro)	6 AD and 6 HC	[18F] PSS232	Increased mGluR5 binding in the frontal cortex (5.2-fold) and hippocampus (2.5-fold) Marginally higher mGluR5 availability (1.2-fold) in cerebellum	Müller Herde et al. [95]
	PET imaging	16 AD and 15 HC	[18F] FPEB	43% reduction of mGluR5 availability in the hippocampus of AD patients.	Mecca et al. [96]
	PET imaging	9 AD and 10 HC	[11C] ABP688	Lower mGluR5 binding in bilateral hippocampus and bilateral amygdala	Treyer et al. [97]
	PET imaging	19 AD and 16 HC	[18F] PSS232	Significantly reduced mGluR5 availability in hippocampus (*p* < 0.001) and parahippocampal gyrus (*p* < 0.001) of AD patients	Wang et al. [98]

## Data Availability

The data that support the findings of this study are available from the corresponding author upon reasonable request.
